# Extensive gene flow of white-backed planthopper in the Greater Mekong Subregion as revealed by microsatellite markers

**DOI:** 10.1038/s41598-017-16164-0

**Published:** 2017-11-21

**Authors:** Yanqiong Yin, Xiangyong Li, Dong Chu, Xueqing Zhao, Khay Sathya, Bounneuang Douangboupha, Mu Mu Kyaw, Manita Kongchuensin, Apirusht Somrith, Vinh Vien Ngo, Huy Chung Nguyen, Shicai Shen, Shufang Liu, Aidong Chen

**Affiliations:** 1 Agriculture Environment and Resources Institute, Yunnan Academy of Agricultural Sciences, Kunming, 650205 China; 2 Key Lab of Integrated Crop Pest Management of Shandong Province, College of Plant Health and Medicine, Qingdao Agricultural University, Qingdao, 266109 China; 3 Plant Protection Office, Cambodian Agricultural Research and Development Institute, Phnom Penh, 01 Cambodia; 4 Horticulture Research Center, National Agriculture and Forestry Research Institute, Vientiane, 7170 Lao PDR; 5 Department of Agricultural Research, Ministry of Agriculture and Irrigation, Nay Pyi Taw, Myanmar; 6 Plant Protection Research and Development Office, Department of Agriculture, Bangkok, 10170 Thailand; 7 Plant Protection Research Institute, Vietnam Academy of Agricultural Sciences, Hanoi, Vietnam

## Abstract

The white-backed planthopper (WBPH), *Sogatella furcifera* (Horváth), is a destructive pest of rice in the Greater Mekong Subregion (GMS) countries including Cambodia, Laos, Myanmar, Thailand, Vietnam, and China’s Yunnan Province. Our previous study not only confirmed the immigration sources of the WBPH in China’s Yunnan Province were from Myanmar, Vietnam, and Laos, but also indicated that Cambodia was likely an additional migration source. To further clarify the migration sources and patterns of the WBPH in the GMS, we investigated the genetic structure of 42 WBPH populations using microsatellite loci markers. The analysis of genetic diversity, heterozygosity deficit, and heterozygosity excess based on the nuclear markers suggest that there is extensive gene flow between the 42 sampled populations from the GMS. The genetic structure confirmed the immigration sources of WBPH as revealed by mitochondrial markers and trajectory analyses methods in previous studies. These findings will aid in the sustainable regional management of this insect pest in the GMS.

## Introduction

The white-backed planthopper (WBPH), *Sogatella furcifera* (Horváth) (Hemiptera: Delphacidae), is one the most destructive pests in rice producing areas of the Greater Mekong Subregion (GMS) including Cambodia, Laos, Myanmar (Burma), Thailand, Vietnam, and China’s Yunnan Province. The migration sources and dispersal patterns within the region have been examined by several researchers in recent years. For example, a number of studies have begun to explore the migration sources of the WBPH based on the trajectory analyses methods^1–6^. Due to their small body size, short lifespan, and long distance dispersal capability, it has been difficult to use fluorescent marker dyes, radar monitoring or other conventional approaches to study the migration of these insects^7^. Molecular makers have the potential to overcome those challenges^8,9^. Using both mitochondrial genes and microsatellite loci as molecular markers, migration patterns of the diamondback moth within China have been identified from the southern to northern regions^10^.

Our previous studies suggested that a small number of the WBPH are able to overwinter in the low latitude paddy area in the southern part of Yunnan Province, China^11^. The immigration sources into Yunnan Province during April to early May were estimated to be mainly from Myanmar, while the mid-May immigrations originate from northern Vietnam^6,12^. Recently, we have used the mitochondrial cytochrome oxidase I (mtCOI) marker to determine the sources and patterns of the WBPH in the GMS^13^, demonstrating that the potential emigration of the WBPH from the GMS consists of three possible major routes. Those results not only confirmed that the immigration sources of the WBPH into China’s Yunnan Province were from Myanmar, Vietnam, and Laos^6,12,14^, but also indicated that Cambodia was a likely additional migration source. As nuclear genetic markers, microsatellite loci have been widely used in elucidating the genetic structure of insect populations, because they are inherited codominantly and have a broad distribution and high abundance throughout the genome^15–18^. Determining the genetic structure of the WBPH in the GMS based on nuclear markers will provide further insights into the gene flow and migration patterns of the WBPH in this region.

In the present study, we investigated the genetic structure of 42 WBPH populations using nuclear (microsatellite loci) markers, to reveal the gene flow and migration patterns of the WBPH in the GMS. These results will benefit future sustainable management programs of this insect pest in the GMS.

## Results

### Genetic diversity based on microsatellite alleles

The data microsatellite locus KJ7 was eliminated due to the existence of the high null allele, thus, only eight loci were used in the analysis of genetic diversity, genetic structure, and gene flow. Values of genetic diversity indexes of the populations from China’s Yunnan Province are given in Table 1. The average number of alleles per locus (*Na*) ranged from 3.375 to 8.625, and the effective number of alleles (*Ne*) ranged from 2.5234 to 5.0602. The expected heterozygosity (*He*) ranged from 0.5639 to 0.7650 while the observed heterozygosity (*Ho*) ranged from 0.2452 to 0.5563. The *He* value in each population was higher than the value of *Ho*. Nei’s expected heterozygosity (*Nei*) ranged from 0.4922 to 0.7459. The level of genetic diversity indexes was similar in most populations.

**Table 1:** Genetic diversity indexes and genetic structure within of *Sogatella furcifera* collections based on microsatellite data.

Collection code	*N*	*Na*	*Ne*	*Ho*	*He*	*Nei*	*Fis*	*Pwil*
JP	25	7.6250	2.9487	0.3465	0.6479	0.6319	−0.0180	**0.0195**
KY	25	6.3750	3.2404	0.329	0.6750	0.6611	**0.0003**	0.2305
YS	25	8.2500	4.5542	0.3614	0.7204	0.7054	**0.1131**	0.4727
FN	25	8.1250	3.7317	0.3478	0.6828	0.6684	0.0372	0.0977
MD	25	6.6250	3.3366	0.4165	0.5872	0.5735	−0.0082	0.2734
BS	25	6.5000	3.4192	0.3715	0.6168	0.6036	−0.022	0.2305
MS	25	6.1250	3.4551	0.3821	0.6242	0.6089	−0.0406	0.2305
YIJ	25	7.5000	4.5493	0.3985	0.6916	0.6765	**0.1402**	0.6289
LC	25	7.6250	4.0353	0.4110	0.6839	0.6700	**0.1404**	0.3203
NE	25	7.0000	3.4736	0.3976	0.6573	0.6430	0.0880	0.0977
MH	25	8.6250	4.7007	0.3504	0.7137	0.6991	**0.0866**	0.1563
SJ	25	7.6250	4.0784	0.3121	0.6779	0.6635	−0.0181	0.3711
GM	25	8.000	4.2825	0.3728	0.6883	0.6728	**0.0895**	0.2734
CY	25	6.8750	3.1437	0.3916	0.6334	0.6202	0.0370	**0.0039**
CX	25	6.2500	3.8024	0.3977	0.6224	0.6068	−0.0419	0.6289
SM	25	7.6250	3.6496	0.2562	0.7041	0.6892	−0.0622	0.1250
XP	25	7.3750	4.1118	0.3614	0.7139	0.6988	0.1045	0.3711
YUJ	25	7.5000	4.3294	0.4152	0.6886	0.6724	**0.1433**	0.1250
ZY	25	8.1250	4.5294	0.3600	0.7221	0.7053	**0.1040**	0.6289
SZ	25	6.3750	3.6770	0.3712	0.6632	0.6496	**0.0498**	0.6289
L1	25	7.2500	3.8766	0.3423	0.6454	0.6320	−0.0266	0.2305
L2	25	7.2500	4.1650	0.3636	0.6307	0.6176	−0.0088	0.4219
L3	25	6.5000	3.8827	0.3019	0.5857	0.5723	−0.1968	0.5273
L4	25	7.0000	3.6879	0.2945	0.6442	0.6299	−0.0937	0.2305
L5	25	7.3750	3.4728	0.2967	0.6425	0.6265	−0.0905	0.1563
L6	25	7.3750	3.8470	0.3485	0.6671	0.6512	**0.0329**	0.2734
L7	25	6.7500	3.7158	0.3562	0.6096	0.5946	−**0.0540**	0.1914
L8	25	8.2500	4.4126	0.2834	0.7222	0.7033	0.0129	0.1914
T1	25	6.8571	3.7785	0.5379	0.6128	0.5786	**0.1293**	0.3438
T2	25	8.2857	4.0453	0.3551	0.7113	0.6910	**0.0882**	0.2891
C1	25	7.8571	3.9816	0.2452	0.7460	0.7303	−**0.0255**	0.3438
C2	25	8.1250	4.1628	0.3467	0.7650	0.7459	**0.1406**	0.2305
C3	25	8.5000	5.0602	0.3653	0.7603	0.7433	**0.1631**	0.5273
C4	25	7.6250	4.4566	0.3085	0.7279	0.7125	**0.0437**	0.9023
V1	25	7.3750	3.9255	0.3478	0.7236	0.7079	**0.0954**	0.3711
V2	25	7.8750	4.7815	0.3072	0.7157	0.6925	0.0222	0.5273
V3	25	7.8571	3.9462	0.3364	0.7167	0.6334	0.0891	**0.0391**
V4	25	5.5000	3.5532	0.3389	0.6984	0.6609	0.0167	0.5273
M1	12	5.6250	3.9255	0.4417	0.6747	0.6421	**0.1747**	0.9609
M2	12	5.7500	4.0031	0.4347	0.6997	0.6612	**0.1990**	0.6797
M3	9	4.1250	3.1872	0.4745	0.6644	0.6181	0.2314	0.9629
M4	5	3.3750	2.5234	0.5563	0.5639	0.4922	0.2225	0.1563

For each sample, the following are indicated: sampling site, population code, date of collection, host plant, sample size (*N*), average number of alleles per locus (*Na*), the effective number of alleles (*Ne*), the observed heterozygosity (*Ho*), the expected heterozygosity (*He*), and Nei’s expected heterozygosity (*Nei*), the estimator of the fixation index (*Fis*), and the Wilcoxon test *P* value for heterozygosity deficit compared to expectations at mutation-drift equilibrium (*Pwil*). Significant values for *Fs* and for heterozygosity deficiency are in bold.

The level of genetic diversity in most populations from different countries was similar. For example, the *He* in Laos populations ranged from 0.5857 to 0.7222 which was similar to those from Thailand (0.6128–0.7113), Cambodia (0.7279–0.7650), Myanmar (0.5639–0.6997), and Vietnam (0.6984–0.7236). The average *He* value in China’s Yunnan populations (0.6707) was similar to those in the adjacent countries (0.6840) (*P* > 0.05) (Table 1).

### Analyses of genetic structure within populations

The estimator of the fixation index, *Fis*, was significantly different in 20 of the 42 populations, demonstrating that the presence of sub-structure within the populations was common (Table 1). In testing for deviation from mutation-drift equilibrium in BOTTLENECK, we detected a significant heterozygosity deficit (Wilcoxon test *P* < 0.05) in only three populations (CY, JP, and V3). The significant heterozygosity deficit in the three populations may result from demographic expansion^18,19^ because there were no significant departures from Hardy-Weinberg equilibrium (*Fis*) in these populations (Table 1), suggesting that the significant deviation from mutation-drift equilibrium was not due to sub-structure (the Wahlund effect) within these localities.

In testing the deviation from mutation-drift equilibrium in BOTTLENECK software, we did not detect a significant heterozygosity excess in any population under the TPM or SMM models, although under the IAM model, a significant heterozygosity excess (Wilcoxon test *P* < 0.05) was detected in twelve of the populations (Table 2), indicating that these twelve populations might have experienced a genetic bottleneck.

**Table 2:** Within-collection tests for heterozygosity excess *P*-values according to three models (IAM, TPM, and SMM).

Collection code	Heterozygosity excess *P*-values		
	IAM	TPM	SMM
CX	**0.03711**	0.42188	0.67969
MD	0.32031	0.76953	0.99414
BS	0.37109	0.80859	0.98633
MS	0.09766	0.80859	0.98633
YIJ	0.09766	0.42188	0.80859
LC	0.42188	0.72656	0.97266
XP	0.09766	0.67969	0.98633
YUJ	0.37109	0.90234	0.98047
NE	0.37109	0.96289	0.99609
MH	0.47266	0.87500	0.90234
SJ	0.15625	0.67969	0.98633
GM	0.27344	0.76953	0.99023
CY	0.57813	0.99805	1.00000
SZ	**0.02734**	0.42188	0.97266
KY	**0.01367**	0.80859	0.99414
YS	0.23047	0.57813	0.90234
FN	0.37109	0.96289	1.00000
JP	0.67969	0.98633	1.00000
SM	0.27344	0.90234	0.98633
ZY	**0.03711**	0.42188	0.98633
L1	0.37109	0.80859	0.98047
L2	0.23047	0.62891	0.97266
L3	0.19140	0.52734	0.97266
L4	0.37109	0.80859	0.96289
L5	0.52734	0.87500	0.99414
L6	0.23047	0.76953	0.99414
L7	0.32031	0.84375	0.98633
L8	0.32031	0.84375	0.99023
T1	0.46875	0.71094	0.96094
T2	0.53125	0.76563	0.99609
C1	**0.02734**	0.71094	1.00000
C2	0.27344	0.80859	0.98633
C3	**0.01367**	0.52734	0.90234
C4	**0.03711**	0.12500	0.87500
V1	**0.00977**	0.67969	0.99023
V2	**0.03711**	0.52734	0.80859
V3	0.28125	0.97656	1.00000
V4	0.23047	0.52734	0.84375
M1	**0.01953**	0.05469	0.65625
M2	**0.03711**	0.37109	0.37109
M3	**0.02734**	0.09766	0.23047
M4	0.87500	0.87500	0.90234

Bold indicates significance at *P* < 0.05.

### Analyses of genetic structure among populations

**Figure 1:**
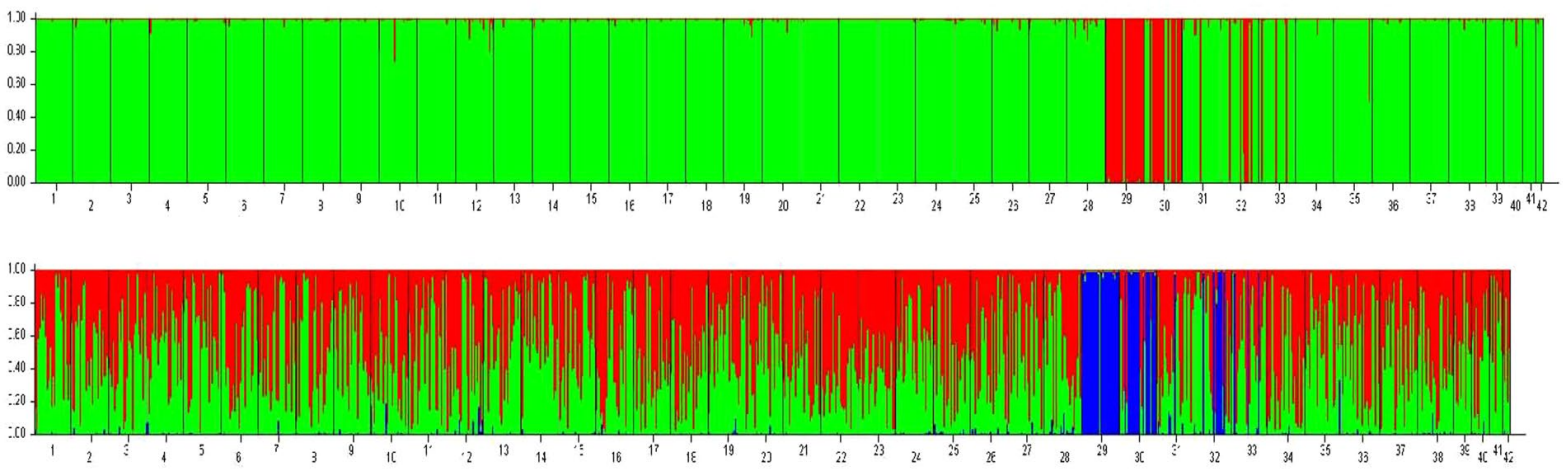
The structure for the microsatellite data set of *Sogatella furcifera* (*K* = 2 and 3, respectively). 1, CX; 2, MD; 3,BS; 4, MS; 5, YIJ; 6, LC; 7, XP; 8, YUJ; 9, NE; 10, MH; 11, SJ; 12, GM; 13, CY; 14, SZ; 15, KY; 16, YS; 17, FN; 18, JP; 19, SM; 20, ZY; 21, L1; 22, L2; 23, L3; 24, L4; 25,L5; 26, L6; 27, L7; 28, L8; 29, T1; 30, T2; 31, C1; 32, C2; 33, C3; 34, C4; 35, V1; 36, V2; 37, V3; 38, V4; 39, M1; 40, M2; 41, M3; 42, M4.

When considering each pairwise *Fst*, 512 of 861 (59.5%) *Fst* values were associated with a significant exact test (Table 3). Analyses using STRUCTURE software identified two genetic clusters overall (*K* = 2) (Fig. 1): one cluster consisted mainly of individuals from the two populations in Thailand (T1 and T2), and a few individuals from three of the populations from Cambodia (C1, C2, and C3); another cluster consisted mainly of all individuals from the other 37 populations, a few of individuals from the two populations in Thailand (T1 and T2), and most individuals from the three populations in Cambodia (C1, C2, and C3). When *K* = 3, the individuals from the two populations from Thailand (T1 and T2), and a few of individuals from three of the Cambodian populations (C1, C2, and C3) could also be differentiated from the other individuals.

**Table 3:**
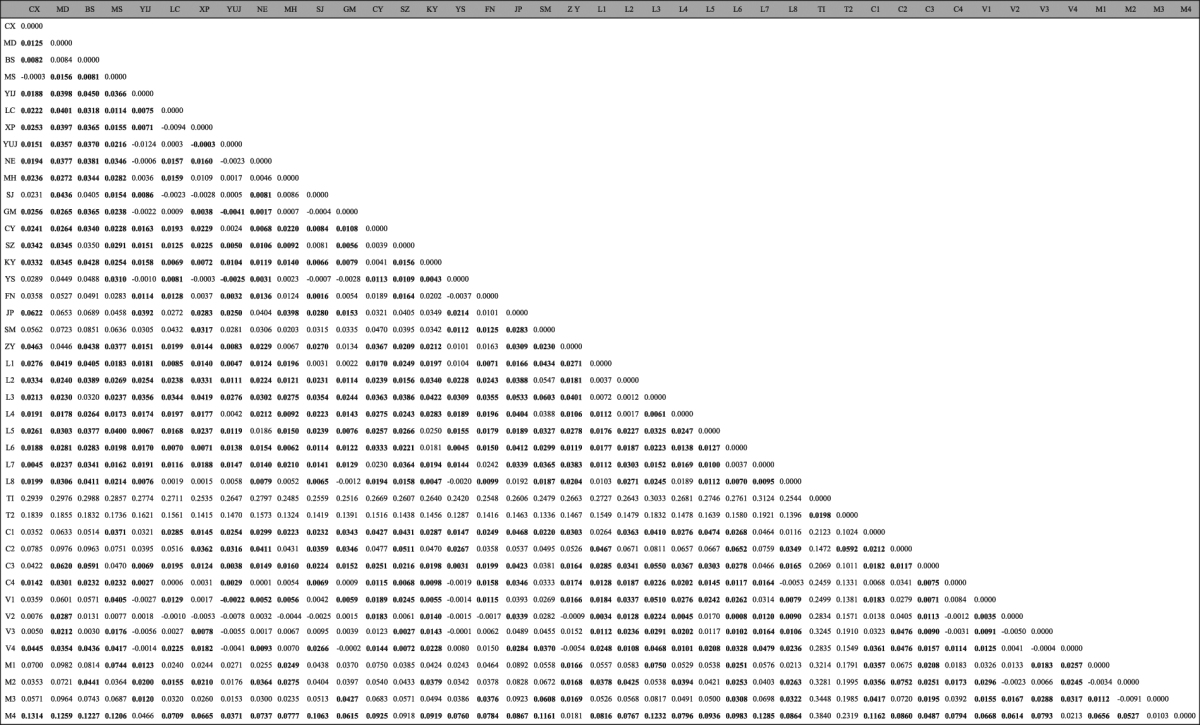
Pairwise *Fst* (genetic distance) between the collections in the Greater Mekong Subregion.

**Figure 2:**
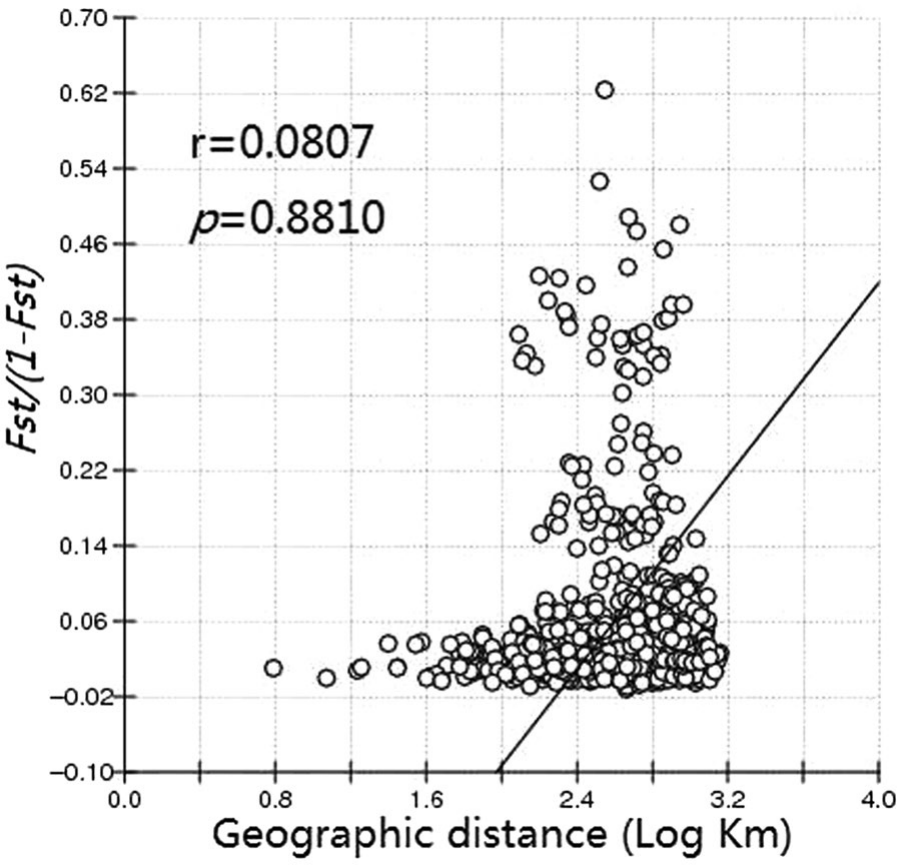
Relationship between genetic distance and log of geographical distance for pairwise population comparisons. *The line represents the regression line and circles represent the logarithm transformation of distance.

The Mantel test results produced an r value of 0.0807 for microsatellite alleles (*P* = 0.8810) (Fig. 2), indicating that no correlations were found between genetic distance and geographical distance among the populations of the WBPH in the GMS countries, indicating that extensive gene flow exists among these WBPH populations.

Significant genetic structure of the WBPH was observed at two hierarchical levels (among populations and within populations) (Table 4). Most of the variation was at the within populations level (91.53%). Although the variation among populations (8.47%, *P* < 0.01) was small, it was significant. These results demonstrated that the variations of genetic differentiation in the WBPH are mainly from inter-populations.

**Table 4:** Analysis of molecular variance (AMOVA) for structures of *Sogatella furcifera* collections.

Source of variation	d.f.	Sum of squares	Variance components	Percentage of variation	
Among collections	41	599.456	0.25373Va	8.47	*P* < 0.001
Within collections	1926	5283.738	2.74337Vb	91.53	
Total	1967	5883.194	2.9971		

### Gene flow based on microsatellite data

Based on microsatellite data, the average values of the numbers of migrants in the different countries were similar except in Thailand (Table 5). In China’s Yunnan Province, the average number of migrants was the highest in southern Yunnan, while the lowest numbers were found in the western region; this is similar to previously published results based on mitochondrial COI data^9^. In Cambodia, Laos, Myanmar (Burma) and Vietnam, the total migrants (*Nem*) ranged from 883.4 (L1) to 3322.1(M4). A high numbers of total migrants (*Nem* > 1000) were found in several populations, including L2, L4-L7 of Laos, C1, C3-C4 of Cambodia, V1-V4 in Vietnam, and M1-M4 of Myanmar, while, Thailand had the lowest number of total migrants, i.e., T1 (*Nem* = 685.9), T2 (*Nem* = 807.7). In Yunnan Province, the total migrants (*Nem*) ranged from 591.5 (CX) to 1224.5 (YUJ). A total of 7 populations had a high numbers of migrants (*Nem* > 1000), including MD in western Yunnan (*Nem* = 1020.0), YUJ and SM in central Yunnan (*Nem* = 1224.5 and1176.0, respectively), GM in southwestern Yunnan (*Nem* = 1182.1), SZ in northeastern Yunnan (*Nem* = 1012.5), FN in southeastern Yunnan (*Nem* = 1207.1), JP in southern Yunnan (*Nem* = 1160.2). Within the province, the number of migrants was the highest in the southeastern region (YS and FN) (average *Nem* = 1071.9), and the lowest in central Yunnan (CX,SM,XP, and YUJ) (average *Nem* = 885.3).

**Table 5:**
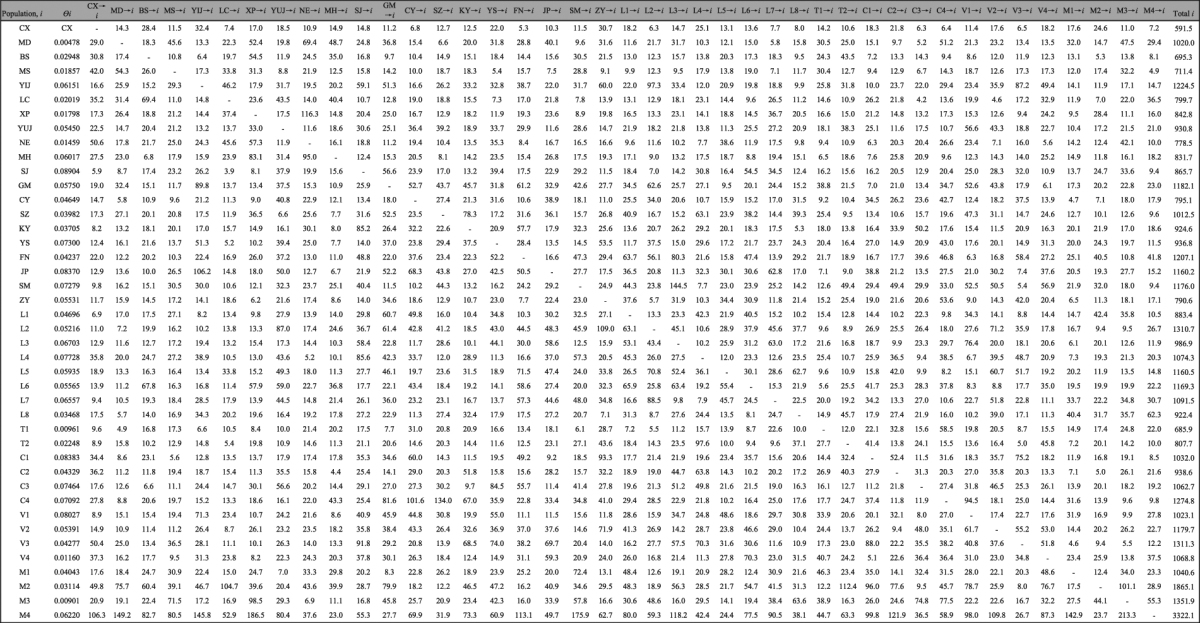
Numbers of effective migrants per generation (*N*_*e*_ *m*) of *Sogatella furcifera*in the Greater Mekong Subregion.

${\boldsymbol{\ominus }}$: mutation-scaled population size, which is effective population size × mutation rate per site per generation; *M:* mutation-scaled immigration rate, which is the immigration rate divided by the mutation rate.

## Discussion

### Evidence for extensive gene flow in the WBPH within the GMS

This study showed that the level of the genetic diversity in most populations originating from different countries was similar, suggesting that extensive gene flow occurs between the WBPH populations within the GMS. The heterozygosity deficit is used to test for population expansion, whereas the heterozygosity excess test is used to provide evidence of a genetic bottleneck. In this study, only three populations from China’s Yunnan Province had a significant heterozygosity deficit. The results in testing the heterozygosity excess were completely inconsistent under the TPM, SMM, and IAM models. Di Rienzo *et al*. showed that most microsatellites fit TPM better than SMM or IAM^20^. Based on the TPM model, there is no significant heterozygosity excess in any of the tested populations, which suggests that no severe bottleneck effects exist in the GMS populations. Our study indicate that bottleneck effects have not played an essential role during the genetic differentiation of the WBPH. This may be due to the bottleneck effects on heterozygosity being transient and observable for only a few generations^21^.

### Migration sources of the WBPH within the GMS

The extensive gene flow between WBPH populations within the GMS is consistent with our previous study^9^ showing that there are multiple immigration sources of the WBPH in China’s Yunnan Province including Myanmar, Vietnam, Laos, and Cambodia. Although the populations from central Thailand (T1 and T2 populations) were shown to have an extensive gene flow in this study, both had limited gene flow with neighboring countries, limiting the probability of immigration of these populations into China’s Yunnan Province. These results support previous results based on the trajectory analyses methods^12,22,23^ that the populations in central Thailand would be incapable of immigrating to Yunnan due to the lack of sufficient wind currents, incorrect wind direction and the excessive distance involved, although gene flow with the populations in Vietnam, Laos, and Cambodia would be possible^23^.

The Cambodian populations (C1-C3 populations, especially C2) do have a limited gene flow with those from central Thailand but have extensive gene flows with other populations from other countries in the GMS. Our previous study showed that the specific mtCOI haplotype from Cambodia is only found outside the country in China’s Yunnan Province (BS and GM populations). The extensive nuclear gene flow also substantiates the probable occurrence. However, the trajectory analyses methods demonstrated that the emigrant population from Cambodia would not be able to migrate to Yunnan Province^23^. The immigration of the WBPH individuals from Cambodia into China’s Yunnan may be indirect from Vietnam or Laos, which have extensive gene flow with those in Cambodia. Whether the immigration of WBPH from Cambodia into China’s Yunnan is direct or/and indirect remains to be determined.

### Future research on the source population of *S. furcifera* within the GMS

The immigration sources and patterns of the WBPH as demonstrated by mitochondrial and nuclear markers are helpful in devising future, sustainable, regional management programs for this important pest in the GMS. However, the genetic basis for the migration should be further explored, including the influence of such factors as wing polyphenism^24,25^. For example, Xu *et al*. showed that two insulin receptors in the migratory brown planthopper (*Nilaparvata lugens*) (Stål) (Hemiptera: Delphacidae) play an important role in controlling long versus short wing development^25^, providing the first evidence of a molecular basis for the regulation of wing polyphenism in insects. We had elucidated the genetic diversity of the WBPH in the GMS countries based on mtCOI and SSR markers. In future studies, it will be necessary to analyze the reliability and significance of these molecular markers relative to their consistency with WBPH biological data. With the exception of the molecular markers, the genome difference and transcriptome analyses also should be considered in a followup study. Although the 42 populations that were collected from the GMS countries help to explain the genetic diversity within somewhat limited areas, additional populations from different regions and from different seasonal occurrence in the GMS should be collected and analyzed in future studies. Additional attention needs to also be paid to more widely distributed populations, such as those from Malaysia, Indonesia, the Philippines, Bangladesh, Pakistan, India and other known occurrences of *S. furcifera* within Asia and outside of Asia to further explore and clarify the source population of *S. furcifera* in the GMS.

## Conclusions

Based on the nuclear (microsatellite) markers, the analysis of the genetic diversity, heterozygosity deficit, and heterozygosity excess suggested that there is extensive gene flow between the WBPH populations in the GMS. The genetic structure confirmed the immigration sources of the WBPH as revealed by mitochondrial markers. There is a certain gene flow between the populations in Thailand and Cambodia. It should be further explored whether the immigration of WBPH from Cambodia into China’s Yunnan Province is direct or/and indirect. These results will be helpful to the sustainable regional management of this insect pest in the GMS.

## Materials and Methods

### Field sampling and DNA extraction

Adult WBPH samples were collected from 42 locations in the GMS during 2014–2015 (Table 6). The samples included 20 populations from China’s Yunnan Province, four from Vietnam, eight from Laos, two from Thailand, four from Cambodia, and four from Myanmar. The specimens were fixed in 95% ethanol and stored at −20 °C until DNA was extracted. Genomic DNA was individually extracted from each adult planthopper using the DNAzol kit (Molecular Research Center, Inc., Cincinnati, OH) and stored at −20 °C.

**Table 6:** Population data of *Sogatella furcifera* populations in the Greater Mekong Subregion (GMS)during 2014–2015.

Regions	Code	Location	Longitude	Latitude	Elevation (meter)	Date*
South Yunnan, China	JP	Jinping, Yunnan	N22.8	E103.2	1349	June 10
	KY	Kaiyuan, Yunnan	N23.5	E103.3	1305.9	June 10
Southeast Yunnan, China	YS	Yanshan, Yunnan	N23.6	E104.3	1579	June 9
	FN	Funing, Yunnan	N23.6	E105.6	680	June 10
West Yunnan, China	MD	Midu, Yunnan	N25.3	E100.4	1653	June 27
	BS	Baoshan, Yunnan	N25.0	E99.1	1699.9	June 26
	MS	Mangshi, Yunnan	N24.3	E98.4	851.2	June 27
	YIJ	Yingjiang, Yunnan	N24.7	E97.9	1682	June 30
	LC	LongChuan, Yunnan	N24.1	E97.7	953	May 27
Southwest Yunnan, China	NE	Ninger, Yunnan	N23.0	E101.0	1312.9	June 16
	MH	Menghai, Yunnan	N21.9	E100.4	1230	May 15
	SJ	Shuangjiang, Yunnan	N23.4	E99.8	1063	July 10
	GM	Gengma, Yunnan	N23.5	E99.3	1116	July 10
	CY	Cangyuan, Yunnan	N23.1	E99.2	1444	June 4
Central Yunnan, China	CX	Chuxiong, Yunnan	N25.0	E101.4	1812.8	June 26
	SM	Songming, Yunnan	N25.3	E103.0	1875.9	July 3
	XP	Xinping, Yunnan	N24.0	E101.9	1502.2	June 17
	YUJ	Yuanjiang, Yunnan	N23.7	E102.0	1202.7	June 17
Northeast Yunnan, China	ZY	Zhaoyang, Yunnan	N27.3	E103.7	1907	July 10
	SZ	Shizong, Yunnan	N24.6	E1042.9	951	June 14
Laos	L1	Hadsayphong District, Vientiane Capital City	N18.2	E102.5	128	March 18
	L2	Thaphabad District, Bolikhamxay Province	N18.4	E103.2	128	March 19
	L3	Bolikhan District, Bolikhamxay Province	N18.3	E103.6	128	March 19
	L4	Hinboun District, Khammouane Province	N17.7	E104.5	130	March 20
	L5	Vapee District, Saravanh Province	N15.6	E105.9	120	March 21
	L6	Saravanh District, Saravanh Province	N15.6	E106.3	223	March 21
	L7	Vapee District, Saravan Province	N15.6	E105.9	119	March 21
	L8	Songkhone District, Savonakhet Province	N16.2	E105.2	115	March 22
Thailand	T1	Nakhon Chum District, Kamphaeng Phet Province	N16.4	E99.4	53	May 14
	T2	Bang Len district, Nakhon Pathom Province	N14.0	E100.2	−11	May 15
Cambodia	C1	Sangkat Prateahlang, Khan Dangkor, Phnom penh	N11.4	E103.2	14	March 24
	C2	Sangkat Dangkor, Khan Dangkor, Phnom penh	N11.5	E104.9	12	March 24
	C3	Stoung District, Kampoug Thom Province	N13.0	E104.5	10	March 27
	C4	Aek Phnum District Battambang Province	N13.3	E103.6	7	March 27
Vietnam	V1	Xuan Linh Commune, Nghi Xuan District, Ha Tinh Province	N18.5	E105.7	10	April 16
	V2	Quang Ninh District, Quang Binh Province	N17.4	E106.6	10	April 17
	V3	Phong An Commune, Phong Dien District, Hue Province	N16.5	E107.3	4.8	April 17
	V4	Phu Loc District, Hue City	N16.3	E107.7	1.4	April 18
Myanmar	M1	Begayet, Ayeyarwady region	N16.8	E94.8	5.5	August 18
	M2	Pathwe, Ayeyarwady region	N17.0	E95.2	2.8	August 19
	M3	Kali, Bago region	N17.3	E96.5	25	August 20
	M4	Kanbaukkyi, Bago region	N18.9	E96.3	60	August 20

^*^all samples were collected in 2014 except those in Myanmar were collected in 2015.

### Microsatellite genotyping and genetic diversity based on microsatellite data

Nine pairs of fluorescent-labeled polymorphic microsatellite primers (KJ6, KJ7, KJ14, KJ16, KJ17, KJ18, KJ25, KJ35, and KJ55) (Table 7) were screened from 40 pairs of newly designed primers based on the WBPH microsatellite sequences in GenBank (until November 11, 2014) and were then used to amplify the loci using WBPH DNA as the template. The primers and the annealing temperature are described in Table 2. The PCR reactions were performed in 20 μL of a solution containing 2 μL 10 × buffer, 1.5 mM MgCl_2_, 0.2 μM dNTPs, 1 unit Taq DNA polymerase, 2 μL template DNA, and 0.2 μM of each primer. PCR amplification was carried out as follows: initial denaturation at 94 °C for 4 min, followed by 35 cycles of 30 s at 94 °C, 90 s at the primer-specific annealing temperature (Table 1) and 60 s at 72 °C, and a final elongation step at 72 °C for 30 min. The products were run on an ABI 3730xl DNA analyzer (Sangon, ShangHai, China) and the allele size was determined by comparing the mobility of the PCR products to the GeneScan™ 400HD size standard using GeneMapper software version 3.2 (Applied Biosystems, ShangHai, China).

**Table 7:** Sequence of microsatellite primers designed in this study.

Code	Motif	Primer sequences	Tm (°C)	Size(bp)
KJ6	(AT)10	F:CAATGGCTGCTTTGATCC R:AACCTCGTCAACAGTCTGTATT	54	298
KJ7	(CGA)5	F:CGCCCGTTCCAATCAATC R:AGGGTCGGTGGGACAAGA	50	212
KJ14	(GTT)6	F:ATGACGCTTCAACACCCA R:AACAAGGCCAAACGAGAc	54	357
KJ16	(AG)10	F:GGATTACTGGATTCGTGCTA R:ACCCTGCTCTAGTCATCTTT	56	271
KJ17	(TGT)7	F:CGCCCGTTCCAATCAATC R:AGGGTCGGTGGGACAAGA	56	186
AG18	AATA)5	F:ACCCGAGCGACCTGATAG R:GCAACCGTTGGACCATTA	59	212
AG25	(TG)7	F:GGGCTGACTGACAAACAT R:CCTCACAGGCACTACACC	56	178
AG35	(TC)10	F:GTTGTGGTGGCGGGCTTAG R:ACAGGCGCTTGAGGATGA	59	160
AG55	(AC)7	F:GACATTGCCCTCGCTTGA R:CTGGACCAACGATGGAACAT	56	127

Based on the microsatellite alleles, the average number of alleles per locus (*Na*), the effective number of alleles (*Ne*), the observed heterozygosity (*Ho*), the expected heterozygosity (*He*), and Nei’s expected heterozygosity (*Nei*) of each of the 42 WBPH populations were calculated using POPGENE v.1.31^26^. The estimator of the fixation index, *Fis*, was performed to detect deviation from neutrality using GENEPOP v.4.2^27^. Wilcoxon test *P* value for heterozygosity deficit compared to expectations at mutation-drift equilibrium (*Pwil*) was calculated using ARLEQUIN v.3.5 software^28^.

### Analyses of genetic structure within populations based on microsatellite data

Deviation of the mutation-drift equilibrium in each population was tested using the BOTTLENECK software^18^. The heterozygosity deficit was evaluated using the Wilcoxon test under the two-phase mutation model (TPM) recommended for microsatellite data^20^. The possibility of bottleneck events within each of the 42 populations was examined under three mutation models [Two Phase Mutation Model (TPM), Infinite Allele Model (IAM), and Stepwise Mutation Model (SMM)], respectively^18,20^. The TPM model was used with default settings of 30% and SMM model, 70%, respectively.

### Analyses of genetic structure among populations based on microsatellite data

The traditional population differentiation approach, Weir and Cockerham’s estimator of the fixation index *Fst* ^29^, was calculated using GENEPOP v.3.4 software^27^. The correlation between genetic differentiation and geographic distance was examined by Mantel test using IBDWS v.3.15 software^28^. The distribution of genetic variation was investigated by the analysis of molecular variance (AMOVA) using ARLEQUIN v.3.5 software^28^, and by calculating allelic diversity, heterozygosity, and pairwise values of *Fst* among the 42 populations. The genetic clustering of samples were examined using STRUCTURE v.2.3.2 software^30^, using the Bayesian clustering approach with a burn-in period of 50,000 iterations and one million Markov chain Monte Carlo (MCMC) repetitions under the admixture ancestry model. Twenty independent runs were performed for each testing *K* value, ranging from *K* = 1 to 42, and Δ*K* was used to calculate the optimal number of genetic clusters (*K*)^31^.

### Gene flow analysis based on microsatellite data

To evaluate the dispersal of the WBPH between the populations in the GMS, the effective numbers of migrants per generation *N*_*e*_ *m* was calculated using microsatellited data respectively. *N*_*e*_ *m* is ${\boldsymbol{\ominus }}$ *M* (${\boldsymbol{\ominus }}$ = *N*_*e*_ μ, where μ is the mutation rate per site per generation; *M* = *m*/μ, where m is the migration rate) calculated using Bayesian search strategies in MIGRATE v. 3.2.16^32^.
